# Determinants of Compliance With Iron and Folate Supplementation Among Pregnant Women in West Iran: A Population Based Cross-Sectional Study 

**Published:** 2018-12

**Authors:** Soraya Siabani, Sina Siabani, Hossien Siabani, Marjan Moeini Arya, Fateme Rezaei, Maryam Babakhani

**Affiliations:** 1 Department of Public Health, Kermanshah University of Medical Sciences, Kermanshah, Iran; 2 Department of Veterinary Medicine, Razi University, Kermanshah, Iran; 3 Department of Health Promotion, University of Sydney, Sydney, Australia

**Keywords:** Compliance, Folate, Iron, Pregnancy, Antenatal Care

## Abstract

**Objective:** To assess the compliance with iron and folate supplementation, and the possibly causally associated factors, among pregnant women in western Iran.

**Materials and methods:** A cross-sectional study of 433 pregnant women, selected randomly amongst those (n = 8,500) attending 40 primary health care centers (PHCCs) in west Iran in 2017. A validated questionnaire was used to gather data, including demographic characteristics, the compliance with iron/folate supplementation and reasons for non-compliance.

**Results:** The participants’ mean age and the duration of their pregnancies when commencing supplementation were (27.86 ± 5.54y [µ ± SD]) and (23.29 ± 9.86w), respectively. The compliance was 71.6% / 28% for iron, and 81.5% / 40% for folate. The commonest causes of poor compliance were forgetfulness and side-effects. Educational status, age, and history of anemia were significantly positively associated with folate compliance. The compliance with iron was associated only with the level of education.

**Conclusion:** Although the compliance with iron and folate was relatively high, most women had not started taking the supplements regularly or at the correct time, usually due to forgetting and/or experiencing adverse side-effects.

## Introduction

Pregnancy is a crucial stage in a woman's life, directly and indirectly affecting the woman and her offspring.Increase in iron and folate requirements due to physiological and hormonal changes in pregnant women, as does fetal demand, leads to an increase in the chance of iron and folate deficiency (1-3). Iron deficiency anemia in pregnancy is a significant problem in developing countries (1-4). 

According to the Center for Disease Control and Prevention, anemia in pregnancy is defined as a hemoglobin level less than 11 g/dl, during the first and third trimesters, or less than 10.5 g/dl in the second trimester (5). Various factors, including malnutrition, incompatibility between needs and intake, parasitic and bacterial diseases, chronic diseases and hematological disorders (e.g. thalassemia) can lead to anemia. 

The commonest cause for anemia in pregnancy would be the incompatibility between needs and intake (6). It is estimated that between 20% and 80% of female in different countries suffer from anemia during pregnancy (6). Anemia can increase the risk of maternal mortality (7), premature delivery, intrauterine growth retardation and low birth weight (8, 9). 

Oral iron therapy is generally effective and convenient for preventing and treating iron deficiency anemia (10). In pregnant women suffering from gastric bypass, uterine bleeding, inflammatory bowel disease or hereditary hemorrhagic telangiectasia, or in those for whom oral iron is ineffective, the intravenous iron is indicated (11).

Pregnancy-related folate deficiency is often associated with megaloblastic anemia, low birth-weight, and congenital anomalies, e.g. neural tube defects (NTDs) (12). NTDs are the second commonest group of congenital anomalies (13). They arise because of a defect in neurulation during embryogenesis– a process occurring 21–28 days after conception – often before women even know that they are pregnant (14-16). 

NTDs are usually associated with other congenital abnormalities which lead to various disabilities and financial burden on the family and society (15). Spina bifida and anencephaly are the commonest NTDs, annually affecting some 300,000 to 400,000 infants worldwide. More serious defects, like anencephaly, are incompatible with lifeand the majority of affected infants do not survive, and those who survive, live with a huge disability (15).

Evidence shows that the taking folate three months prior to neurulation can reduce the risk of NTDs by 75% (14, 12, 15, 17). Pregnant women in low- and middle-income countries, suffering from baseline malnutrition, must start iron and folate supplementation therapy before the appropriate stage of their pregnancy and then continue it throughout the pregnancy (12). 

The World Health Organization (WHO) recommends that all pregnant women should take a daily dosage of 30–60 mg of elemental iron (The equivalent of 30 mg of elemental Iron is 150 mg Ferrous Sulfate heptahydrate, 90 mg ferrous fumarate or 250 mg Ferrous gluconate) and 400µg (0.4 mg) folate as part of their antenatal and neonatal care. This could improve pregnancy outcomes and prevent iron and folate deficiency (3, 16). This recommendation is for women with normal hemoglobin. The time for commencing regular supplementation is different for anemic women, and their anemia should be treated before pregnancy, otherwise, WHO recommends extra supplementation throughout pregnancy.

Nowadays, although iron/folate supplements are provided free or cheaply in most countries, the major problem is noneor poor compliance. This might be due to personal behaviors, cultural issues, and environmental factors, lack of awareness, socio-demographic status, economic factors, inadequate service delivery or side-effects (18, 19). Unpleasant side-effects of iron can be constipation, diarrhea, gastric cramping, nausea, vomiting, metallic taste and blacks tools (11, 18). 

In Iran, where iron and folate are provided free by primary health care centers for all pregnant women, some studies have demonstrated suboptimal compliance with iron/folate supplementation. 

On the other hand, the Iranian government, which concerned about the recent reduction in the fertility rate, has encouraged women to have three or more children. This policy might increase the incidence of pregnancy-related problems, including iron/folate deficiency.

Therefore, noneor poor compliance with iron/folate supplementation and any possibly causative associated factors must be examined whether prevention is to be addressed appropriately. The current study was conducted among pregnant women in western Iran during 2016 and 2017, to assess the compliance with iron/folate supplementation and the factors likely affect the compliance.

## Materials and methods

Kermanshah, in western Iran, population 1,952,433 (964419 women), is a capital city with many disadvantaged people. More than 40 Primary Health Care Centers (PHCCs) provide free primary care services, especially to women and children. Cared-for pregnant women were selected for our study.

433 pregnant women selected to participate in this cross-sectional study. We chose this number because two similar studies had reported a compliance rate of 50% (20) and 40% (1) and, using a confidence level of 95% and a marginal error of 5%, the computed sample size was 385. On the other hand, based on a Cochrane table for determining sample size, the minimum was 379. However, considering a possible 10-15% non-response rate, a sample size of 433 was considered appropriate. 

In order to select the participants, we used a three-step sampling technique: cluster sampling, proportional and randomization. First, 10 PHCCs (each one a cluster) were randomly selected among 40 PHCCs. Then, the sample size for each PHCC was considered. This was based on its population coverage (the number of pregnant women receiving primary care services there). In the last step, the pregnant women registered with each PHCC were randomly selected using Excel randomization.

Participants were excluded from the study if they: 

     (i) were non-residential(living in Kermanshah city less than 6 mounts), and/or 

     (ii) were in the last month of pregnancy, and/or 

     (iii) did not formally consent to participate.

The eligible pregnant women, that signed consent forms, were interviewed individually by two trained interviewers using a questionnaire based on the study’s objectives. The validity and reliability of the questionnaire had been tested and established, using a pilot study with 30 participants and obtaining experts' opinions, including three midwives and four gynecologists. The questionnaire comprised three parts:

1. Socio-demographic information, obstetric and medical characteristics: age, educational status, employment, economic characteristics (including family income, house ownership, and living status – see Table1), gestational age, the number of parity, the interval between the recent and previous pregnancy (if any) and past medical history.
2. The compliance with iron and folate supplementation, including separate consumption of each: Do you take folate/iron supplements? When did you start them? How do you take them?
3. Causes of likely non-compliance with each of the supplements.

The interviewers had been trained to ensure that the participants completely understood their word. In addition, in order to increase the accuracy of answers, caregivers working in the PHCC were asked to answer the same questions regarding each participant's compliance. For non-compliers, further questions were asked through an open-ended questionnaire. After collecting the data, the results were categorized into eight categories, as listed in table 3. 

Data analysis was performed using the statistical package for social sciences (SPSS), version 21. Normally distributed variables (e.g., age and gestational age) were described using mean ± standard deviation (µ ± SD). The Chi-square test was used to assess any association between the compliance and possibly explanatory factors. A probability value (p-value) of less than 0.05 was considered statistically significant. 

The Research Ethics Committee at Deputy of Research of the Kermanshah University of Medical Sciences (KUMS) had approved the study protocol and had monitored the research process. Further, the participants had been given the participant information statement and had signed the written consent form. Individual personal information was kept confidential.

## Results

All 433 pregnant women completed the interviews (Table 1). 

**Table 1:** Sociodemographic, economical, and medical information of studied pregnant women in 2016-17 (n = 433)

**Characteristic**	**Subgroup**			**N (%)**	**Characteristic**	**Subgroup**	**n (%)**
Age	21-30			244(56.4)	Taking Iron supplement	Before conceiving	88(20.3)
	31-35			105(24.2)		With pregnancy	144(33.3)
	36 ≤			34(7.9)		3-4 month after pregnancy	124(28.6)
Educational status			Illiterate	4(0.9)	Iron supplement type	Ferrous sulfate pill	220(50.8)
	Primary/guidance school			119(27.5)		Hematinic pill	16(3.7)
	High school			172(39.7)		Phenols capsules	88(20.3)
	College& university			138(31.9)	Taking folate supplement	Before conceiving	175(40.4)
Work status	Housewife/ unemployed			391(90.3)		With pregnancy	170(39.3)
	White collar			29(6.7)	Parity	prim gravida	221(51.0)
	Other			11(3.0)	Preceding pregnancies	1-3 years earlier	58 (13.4)
Family income	5 × 10^5^ -10 × 10^5^ T			190(43.9)		4 or more years earlier	154(35.6)
	10 × 10^5^ -20 × 10^5^ T			206 (48.0)	Antenatal care (ANC)	Regular	371(85.7)
	20×10^5^ T ≤			61 (14)	Healthcare access	PHCC(only)	87(21.1)
House ownership		Tenant		242(55.9)		PHCC and gynecologist	340(78.5)
	Landlords			173(40.0)	Prior low birth weight	-	14(3.2)
Prior chronic illness		-		91(21.0)	premature birth	-	37(8.5)
Prior anemia	-			50(11.5)	Gestational age	20≤	184(42.5)

N, Number;T, Tooman (Iranian Currency).

**Table 2:** Frequency of personal characteristic and their association with adherence to folate/iron

**Factors**	**Compliance with Iron**				**Compliance with folate**			
	**Yes (%)**	**n (%)**	**Total (%)**	**P-value**	**Yes (%)**	**n (%)**	**Total (%)**	**P-value**
Age					236(80.6)			
21-30	212 (72.1)	82 (27.9)	294 (68.3)	0.41	86(81.9)	57(19.4)	293(67.9)	0.01
31-35	71(67.6)	34(32.4)	105(24.2)		31(91.2)	19(18.1)	105(24.2)	
36≤	26(76.5)	8(23.5)	34(7.9)			3(8.8)	34(7.9)	
Educational status					1(25.0)			
Illiterate	1(25.0)	3(75.0)	4(0.9)		94(79.0)	3(75.0)	4(0.9)	
Primary/guidance school	80(67.2)	39(32.8)	119(27.5)	0.1	141(82.0)	23(19.3)	119(27.5)	0.02
High school	28(74.4)	44(25.6)	172(39.7)		117(84.8)	31(18.0)	172(39.7)	
College and university	100(72.5)	38(27.5)	138(31.9)			21(15.2)	138(31.9)	
Family income					190(84.2)			
5×10^5^ -10×10^5 ^T	128(67.4)	62(36.2)	190(43.9)	0.09	182(77.5)	30(15.8)	160(43.9)	0.47
10×10^5^ -20×10^5^ T	138(75.8)	44(24.2)	182(42.0)		61(78.2)	39(21.4)	141(42.0)	
20×10^5^ T ≤	43(70.5)	18(29.5)	61(14.1)		50(11.5)	9 (12.8)	70(14.1)	
Prior anemia	34(68.0)	16(32.0)	50(11.5)	0.34		14(28.0)	35(70.0)	0.03

Their mean age and gestational stage of their pregnancies were 27.86 ± 5.54 (µ ± SD) years and 23.29 ± 9.86 weeks, respectively. 244 participants (56.4%) were aged between 21 and 30. 39.7% had completed high school (Table 1). Most (90.3%, 391/433) were unemployed or housewives (Table 1). The mean family monthly income was 15,328,200 ± 10,599,800 Rials which corresponds with $320 – with a median income of 15,000,000 Rials. About 56% were tenant and 40.0% landlord (Table 1). 

Thirty-three percent had started taking iron supplements immediately when their pregnancy was diagnosed. More than 28% reported taking iron supplementation according to the recommended time (3-4 months after starting the pregnancy) (Table 1). Ferrous sulfate pills were the most commonly used iron supplement (50.8%).

40.4% started taking folate before conceiving. 39.3% reported taking folate supplementation from the date of the positive pregnancy test. 

51% of the participants were primigravida. Preceding pregnancies in 154 other participants (35.6% of the total) had occurred 4 or more years earlier (Table 1). 

371 (85.7%) women had been receiving regular antenatal care during their current pregnancy. About 78.5% had received primary care from both a PHCC and a gynecologist (Table 1). The history of premature birth and low birth weight were 8.5% and 3.2% respectively. 11% of the participants had a history of pregnancy anemia. About 20% had a history of a major chronic illness, such as hypertension, heart diseases, diabetes, rheumatoid arthritis, anemia, asthma or epilepsy (Table 1). 

The compliance with iron supplementation was 71.6% and with folate 81.5%. The caregivers, who had been asked the same questions, reported 72.3% compliance for iron and 81.3% for folate. There was no significant difference between the self-reported results and the caregivers’ reports (p-value = 0.09). Table 2 presents the association between the compliance with iron/folate supplementation and personal characteristics. Educational status, age and a history of anemia were associated significantly with the compliance with folate supplementation (p-value ≥ 0.05).

The two main reasons for not taking pills were: (i) forgetting to take the pills and (ii) experiencing side-effects such as gastrointestinal problems (Table 3).

**Table 3:** The reasons for poor compliance with iron and folate supplementation

**Barrier **	**Iron supplement (n = 123)**	**Folate supplement (n = 84)**
Forgetting to take the pills most days	69 (56.25)	54 (67.5)
Being unaware about the benefits	5 (4.0)	2 (2.5)
Experiencing side-effects	36 (29.16)	12 (15.0)
Concerning about being harmful	3 (2.08)	2 (2.5)
Getting advice from people	-	4 (5.0)
Not afford of purchasing supplements	5 (4.0)	2 (2.5)
No believe in supplements usefulness	3 (2.08)	2 (2.5)
Growing tired of taking pills	3 (2.08)	-

## Discussion

Our study examined the compliance with iron and folate supplementation and possibly associated causative factors among pregnant women in west Iran. In general, the compliance was relatively high compared with the results of some other studies. However, 20% of a large number of pregnant Iranian women is a considerable number of women, and attention should be paid to non-compliance. Furthermore, the time of starting supplementation was not optimal. 

Ratanasiri and colleagues reported 73% total compliance with iron and folate supplementation in Nepal in 2014 (20). Likewise, Seck and Jackson showed 69% compliance with iron supplementation in Senegal in 2007 (21). An 81.4% compliance with folate supplementation was reported by Riazi and Bashirian, in Hamadan State in Iran. Hamadan is a state closed to Kermanshah but people living there have a different culture in terms of nutritional habitats and lifestyle from those living in Kermanshah (22). 

A study by Ibrahim et al. showed 41% compliance with iron/folate supplementation in Ismailia 2008(1). Gebre's survey in Tigray, Ethiopia, reported about 37% compliance with iron/folate supplementation in urban communities and 29% compliance in rural communities (23). A 50% iron compliance rate was reported by Habib and colleagues in Riyadh, Saudi Arabia (24). Passarelli et.al reported 42% compliance with folate supplementation in Sao Paolo, Brazil 2014 (25). 

Regarding the starting date of supplementation, our findings show that women in western Iran had not followed an optimal schedule. According to Iranian guidelines, they should take a daily dose of 30–60 mg of elemental iron from the 4th month of pregnancy (16th week of gestational age) until 3 months postpartum. They should also take a daily dosage of 400 µg (0.4 mg) folate from the date they plan to be pregnant (three months or at least one month before pregnancy) until 10 to 12 weeks postpartum. The results of our study show that less than one-third of the participants had started iron at the optimal time, though they had done better about starting folate (40%).

Our results demonstrated that the level of women’s education, their age, and the history of anemia was significantly positively associated with the compliance with folate supplementation. These results are consistent with the findings of Passarelli et al. (25) and García-Fragoso et al., (26) who showed that a higher level of education had a positive association with the compliance with folate supplementation. This difference between educated people and non-educated people might be explained by the possible effect of education on self-care skills; as the better-educated are more likely to understand and meet their own needs (27). Similarly, Ogundipe et al. (28) and Gebre1 et al. (23) reported that women with a history of anemia during their previous pregnancy are more likely to perceive the benefit of supplementation during a subsequent pregnancy and to appreciate the dangers of non-compliance. 

Our study showed that age has a positive association with the compliance with folate. An earlier study in Nepal reported a similar relationship between age and compliance with supplements (29). However, by contrast, in a study conducted in Tigray, Ethiopia, age was a negative predictor for the compliance with folate supplementation (23). 

On the other hand, we found no significant association between family income and the compliance with both supplementations. This might be due to easily accessible free primary care services – maternity care in particular. Additionally, pharmacists dispense folate and iron with low co-payments. 

The participants mentioned several reasons for not complying: 

1. Forgetfulness: This could be addressed with better counseling during antenatal visits. Providing strategies reminding women to take their pills on time (e.g. placing the tablets in a site they see every day) might help (24). In studies in Iranshahr (south Iran) (30) and Semnan (central Iran) (31), forgetfulness was the main factor.

2. Adverse side-effects of the iron:A lot of participants experienced gastrointestinal side-effects. This was in line with other studies (11). Two similar studies, in Nepal (19) and Nigeria (28), showed that side-effects were the main barriers to adherence. Although these side-effects are transient and not harmful, we recommend providing supplements without unpleasant side-effects. Educating women about the benefits of supplementation and managing side-effects would be useful, for instance, those who get stomach upsets or pain should be advised to take the iron supplement with food (19).

Our study faced three limitations:

- We used self-reported data to assess the compliance; such data might be less accurate compared with observation. To counter this limitation and to assess the accuracy of the responses, we asked caregivers the same questions about compliance.
- The nature of the study design (cross-sectional) did not allow further evaluation of any apparent associations over time.

We did not assess the format of the different iron supplements, nor the specific side-effects for each format.

## Conclusion

In conclusion, although the compliance with iron/folate in pregnant women in western Iran was relatively high, most had not started at the optimal time. Level of education is a significant predictor for the compliance. Hence, health-care providers and health educators should focus on a standard starting time during prenatal care programs. Further, the compliance with iron and folate in pregnant women can be promoted by minimizing the side-effects of the iron. Providing a strategy for reminding women to take their pills on time would improve adherence.
